# Ice Recrystallization Inhibition Is Insufficient to Explain Cryopreservation Abilities of Antifreeze Proteins

**DOI:** 10.1021/acs.biomac.1c01477

**Published:** 2022-01-26

**Authors:** Yuling Sun, Daria Maltseva, Jie Liu, Theordore Hooker, Volker Mailänder, Hans Ramløv, Arthur L. DeVries, Mischa Bonn, Konrad Meister

**Affiliations:** † Max Planck Institute for Polymer Research, 55128 Mainz, Germany; ‡ Institute of Chemistry, Chinese Academy of Sciences, Beijing 100190, P. R. China; § University of Alaska Southeast, Juneau, Alaska 99801, United States; ∥ Dermatology Department, University Medical Center of the Johannes Gutenberg-University, 55131 Mainz, Germany; ⊥ Roskilde University, 4000 Roskilde, Denmark; # University of Illinois at Urbana−Champaign, Urbana, Illinois 61801, United States

## Abstract

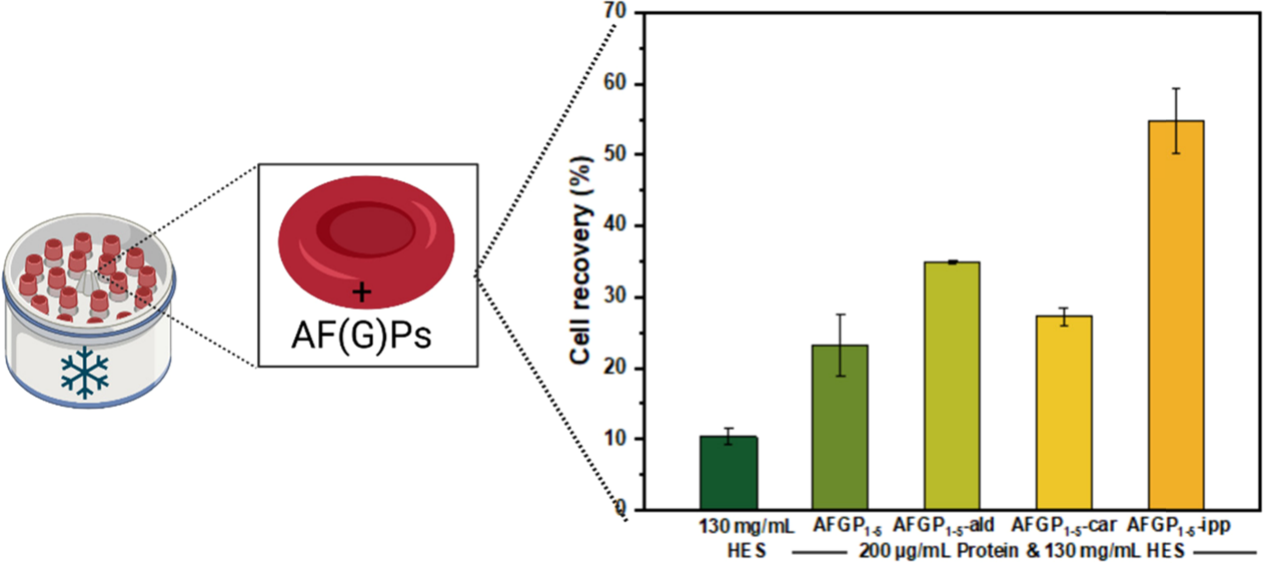

Antifreeze proteins (AFPs) and glycoproteins (AFGPs) are exemplary at modifying ice crystal growth and at inhibiting ice recrystallization (IRI) in frozen solutions. These properties make them highly attractive for cold storage and cryopreservation applications of biological tissue, food, and other water-based materials. The specific requirements for optimal cryostorage remain unknown, but high IRI activity has been proposed to be crucial. Here, we show that high IRI activity alone is insufficient to explain the beneficial effects of AF(*G*)Ps on human red blood cell (hRBC) survival. We show that AF(*G*)Ps with different IRI activities cause similar cell recoveries of hRBCs and that a modified AFGP variant with decreased IRI activity shows increased cell recovery. The AFGP variant was found to have enhanced interactions with a hRBC model membrane, indicating that the capability to stabilize cell membranes is another important factor for increasing the survival of cells after cryostorage. This information should be considered when designing novel synthetic cryoprotectants.

## Introduction

The transfusion of human red blood cells (hRBCs) is oftentimes the only option for patients suffering from leukemia, anemias, or traumas with severe blood loss.^1,2^ Long-term storage of RBCs is further crucial for the storage of rare blood cells, military transfusions, or special autotransfusion programs.^3^ Cryopreservation still remains the only method that allows long-term storage of hRBCs and access to quantities of hRBC units that are necessary when large numbers of hRBC transfusions are required.^4^ Unwanted ice formation and recrystallization during thawing present major challenges for cryopreserved biological samples and cause cellular damage of tissues and hRBC apoptosis.^5,6^ The clinical strategies for cryopreservation require the addition of high concentrations of cell-permeating cryoprotectants such as water-miscible organic solvents (e.g., glycerol and dimethyl sulfoxide).^7−9^ However, the high concentration of cryoprotectants and the toxicity of solvents require time-consuming removal of traces of toxic solvents, conflicting with rapid transplant or transfusions. Furthermore, the current cryopreservation protocols do not effectively control extracellular ice growth, leaving tissues vulnerable to cellular damage.^7,10,11^ Organisms inhabiting freezing environments and prone to ice recrystallization (IRI) injuries have evolved biomolecular solutions to enable life to flourish under icy conditions.^12−14^ They produce antifreeze proteins (AFPs) and glycoproteins (AFGPs) that have the ability to inhibit IRI,^15^ the ability to shape ice crystals into unusual morphologies,^16^ and the ability to depress the freezing point in a noncolligative manner (thermal hysteresis, TH).^17^ The relative magnitude of each effect varies between individual AF(*G*)Ps, with the AFGPs being the by far most potent IRI inhibitors.^16,18^ High IRI activity has been suggested to be key for optimal cryopreservation effects, and substantial efforts have been made to develop synthetic compounds that mimic AF(*G*)Ps IRI properties to increase cellular survival.^5,10,19,20^ However, up to now, the most active synthetic IRI compounds are substantially less active than their natural counterparts, and antifreeze mimics are rarely used in real-life applications. Recently, numerous studies have reported hydroxyethyl starch (HES) as a promising nonvitrifying cryoprotectant of RBCs,^21,22^ peripheral blood stem cells,^23,24^ keratinocytes,^25−27^ fibroblast,^26^ and other cell types.^28,29^ When used at appropriate concentrations, HES appears free of side effects and is less toxic than the commonly used cryoprotectants.^30−32^ However, transfusions containing a high concentration of HES can also have unwanted side effects like the inhibition of hemostasis in vulnerable patients.^33,34^

The usage of AF(*G*)Ps to protect RBCs has been shown to reduce the destruction of RBCs, hemolysis, after cryopreservation.^35^ Carpenter et al. showed that upon the addition of AFP I to RBCs cryopreserved in HES, their recovery due to the inhibition of IRI was enhanced.^11^ Interestingly, it was further reported that AFPs type I, II, and III reduced hemolysis at micromolar concentrations while enhancing hemolysis at AFP millimolar concentrations.^36^ The origin of this discrepancy, the specific requirements for optimized cryopreservation of AF(*G*)Ps and mimics, and the role of IRI activity in cryopreservation remain debated. Here, we investigate the effects of different AF(*G*)Ps and variants on the survival of hRBC after cryostorage. Our results reveal that IRI activity alone is insufficient to explain the cell recovery after cryopreservation and that favorable protein-membrane interactions seem important.

## Materials and Methods

### Human RBCs Preparation

Human blood was collected from healthy donors and centrifuged (1940*g*, 5 min, 25 °C). The top layer (buffy coat and plasma) was removed and replaced with an equal volume of PBS buffer (Dulbecco’s phosphate-buffered saline, 1×, without calcium and magnesium). The RBCs were then washed three times with PBS buffer using identical conditions. The final PBS solution of RBCs had a packed cell volume of ∼40%. Human blood was obtained from the Department of Transfusion Medicine Mainz from 10 healthy donors after physical examination and after obtaining their informed consent in accordance with the Declaration of Helsinki. The use of human blood was approved by the local ethics committee “Landesärztekammer Rheinland-Pfalz” (837.439.12 (8540-F)).

AFGP_1–5_ was purified from the Antarctic toothfish *Dissostichus mawsoni*, as described previously.^28^ The AFGP_1–5_ variants were made by established protocols, as described recently.^37^ AFP I was purified from winter flounder, *Pseudopleuronectes americanus*. RmAFP was obtained by recombinant protein expression, as described elsewhere. Quaternary aminoethyl (QAE) (AFP-III) was purified from the Antarctic eelpout (*Lycodichthys dearborni*) or obtained by recombinant protein expression, including the mutant T18N. In this mutant, the threonine residue at position 18 is replaced by asparagine, which causes the complete loss of antifreeze activity.

### Cryopreservation of RBCs

Freshly prepared human RBCs (50 μL) (packed cell volume ∼40%) were added to 50 μL of cryoprotectant (AFGP_1–5_, AF(*G*)Ps/HES, or AF(*G*)Ps variants/HES) in cryovials and was gently mixed using a vortex. Triplicate independent samples were characterized for each group. All samples were rapidly frozen in liquid nitrogen and stored in liquid nitrogen for 20 min. Samples were thawed for 10 min in a 45 °C water bath or for 20 min at room temperature (22 ± 1 °C).

Control samples (0% hemolysis) were prepared by adding 50 μL of freshly prepared hRBC to PBS buffer and storing them for 1 h at room temperature. Control samples (100% hemolysis) were prepared by adding 50 μL of freshly prepared hRBC to 50 μL of milli-Q water, and samples were vortexed. The 100% hemolysis samples were rapidly frozen in liquid nitrogen and stored in liquid nitrogen for 20 min. Samples were thawed for 10 min in a 45 °C water bath or for 20 min at room temperature (23 ± 1 °C).

### Measurement of RBC Hemolysis and Cell Recovery

Sixty microliters of RBC/cryoprotectant suspension were added into 540 μL of PBS solution and centrifuged (500*g*, 5 min, 4 °C). Thereafter, 200 μL of the supernatant was removed and added into 3.8 mL of PBS solution. Absorbance was measured with an ultraviolet/visible (UV/vis) spectrometer (Lambda 900, Perkin Elmer) at 414 nm (1 cm light path). Hemolysis (%) and cell recovery (%) were calculated according to eqs 1 and 2, respectively. Triplicate samples were characterized for each group.
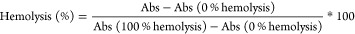
1
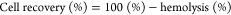
2The Abs (0% hemolysis) and Abs (100% hemolysis) represent the 414 nm absorbance values of the 0% and 100% hemolysis and control samples.

### Preparation of the RBC Model Membrane

The phospholipids 1,2-dioleoyl-sn-glycero-3-phosphocholine (DOPC), N-palmitoyl-d-erythro sphingosylphosphorylcholine (SM), and 1,2-dioleoyl-sn-glycero-3-phosphoethanolamine (DOPE) (Avanti Polar Lipids) were dissolved in chloroform/methanol (9:1 mixture (vol/vol)) at a ratio of 45:45:10 (mol %). This composition was reported to serve as a phospholipid model of the outer leaflet of human RBC membranes.^38^

### Surface Pressure Measurements

Surface pressure measurements were performed in a custom-made Teflon trough filled with 5 mL of PBS buffer at room temperature (22 ± 1 °C) using a DeltaPi tensiometer (KBN 315 Sensor Head, Kibron Inc.). The surface pressure experiments were also performed under a nitrogen atmosphere to prevent the oxidation of unsaturated lipids.^39,40^ Surface pressure measurements were first performed at the air/buffer interface, and the phospholipid mixture solution of DOPC/SM/DOPE was spread droplet by droplet at the air/ buffer interface using a Hamilton syringe.^41,42^ AFGP_1–5_ (200 μL), AFGP_1–5_-ipp (5 mg/mL in PBS), or pure buffer was injected into the subphase. The bulk AFGP concentration was 0.2 mg/mL.

## Results

The cryopreservation of hRBCs was evaluated using a rapid freezing protocol, following experimental procedures described in established protocols.^7,10^ Samples were rapidly frozen by immersion in liquid nitrogen and subsequently stored at −196 °C. Cell recoveries were determined after thawing at an ambient temperature of 23 °C or at 45 °C.

**Figure 1:**
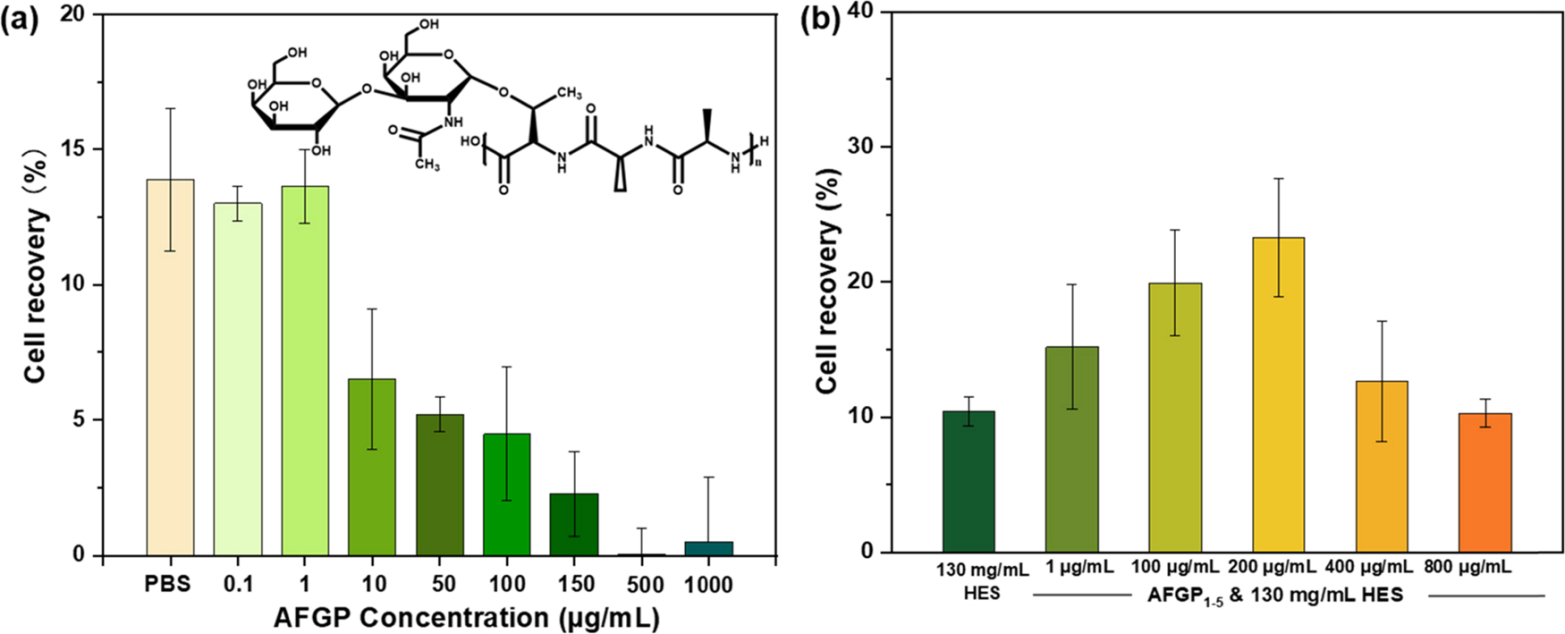
Cryopreservation of hRBC in the presence of AFGP_1–5_. (a) Recovery of hRBC cryopreserved in PBS solutions with different concentrations of AFGP_1–5_ thawed at 45 °C. (b) Recovery of hRBC cryopreserved in HES solutions (130 mg/mL) with different AFGP_1–5_ concentrations thawed at 23 °C. Experiments were performed three times, and the error bars represent the standard deviation between the individual measurements.

Figure 1a shows the results of the hRBC cryopreservation experiment in PBS buffer. The cell survival of hRBCs in PBS buffer was found to be low, with a recovery of ∼14%. Upon the addition of AFGP_1–5_, the cell recovery decreased with increasing AFGP_1–5_ concentration. We found that for AFGP_1–5_ concentrations exceeding 150 μg/mL, the hRBC cell recovery was only ∼2%. Clearly, the presence of AFGP_1–5_ in PBS buffer caused more damage than protection to the hRBC. This finding is in line with the results from previous RBC cryopreservation studies using AFP type I and III.^11,36^ AFGPs can bind to ice crystals^43^ and shape small ice crystals to blunt hexagonal bipyramid crystals at a temperature slightly below its melting point. Upon lowering the temperature below the hysteresis gap, an ice crystal in the presence of AFGPs will rapidly grow into a bundle of spicular ice needles that likely damage the cells.^44^ During the rapid freezing and thawing process, which mimics the cryopreservation of RBCs, bipyramidal,^11^ needle-like, and specular ice crystals^45^ were observed in the presence of AFPs, which damaged the RBCs and reduced cell survival significantly. Upon increasing the AFGP_1–5_ concentrations in PBS buffer, this effect will become increasingly prominent, likely leading to the observed lower cell recovery of hRBCs.

Next, we investigated the capacity of AFGP_1–5_ to increase the survival of hRBC in HES solutions. We find that low (130 mg/mL) and high (215 mg/mL) concentrations of HES resulted in ∼12 and ∼78% hRBC recovery (Figures 1b and S1), respectively, and these cell recoveries are consistent with previous studies.^10^ Compared with AFGP_1–5_ in PBS buffer, which caused significant damage to hRBCs, AFGP_1–5_ in HES solution exhibits much better protection owing to the suppression of the explosive ice growth beyond the TH gap.^44^ Even higher cell recovery rates can be obtained for AFGP_1–5_ in high HES concentrations (215 mg/mL), but the very high HES concentration also leads to very viscous solutions with high osmotic pressure, which is impractical for clinical cryopreservation.^44,46^ In addition, transfusions containing high concentrations of HES have been shown to cause unwanted side effects like the inhibition of hemostasis in vulnerable patients.^33^ Therefore, the ability of AFGPs to modulate cellular recovery at low HES concentrations (130 mg/mL) will be investigated.

Figure 1b shows the results of the cryopreservation experiment of AFGP_1–5_ in a 130 mg/mL HES solution. The effect of AFGP_1–5_ on the hRBC cryopreservation survival was again dependent on the AFGP_1–5_ concentration. We find that the cell recovery of hRBCs increased for concentrations up to 200 μg/mL AFGP_1–5_ but decreased again at higher concentrations. The addition of 200 μg/mL AFGP_1–5_ showed the highest cell recovery (∼24%) with twice the cell recovery of 130 mg/mL HES alone. The subsequent decrease in the cell recovery at higher concentrations agrees with observations for PVA,^10^ AFGP analogs,^47^ and AFP I.^11^

**Figure 2:**
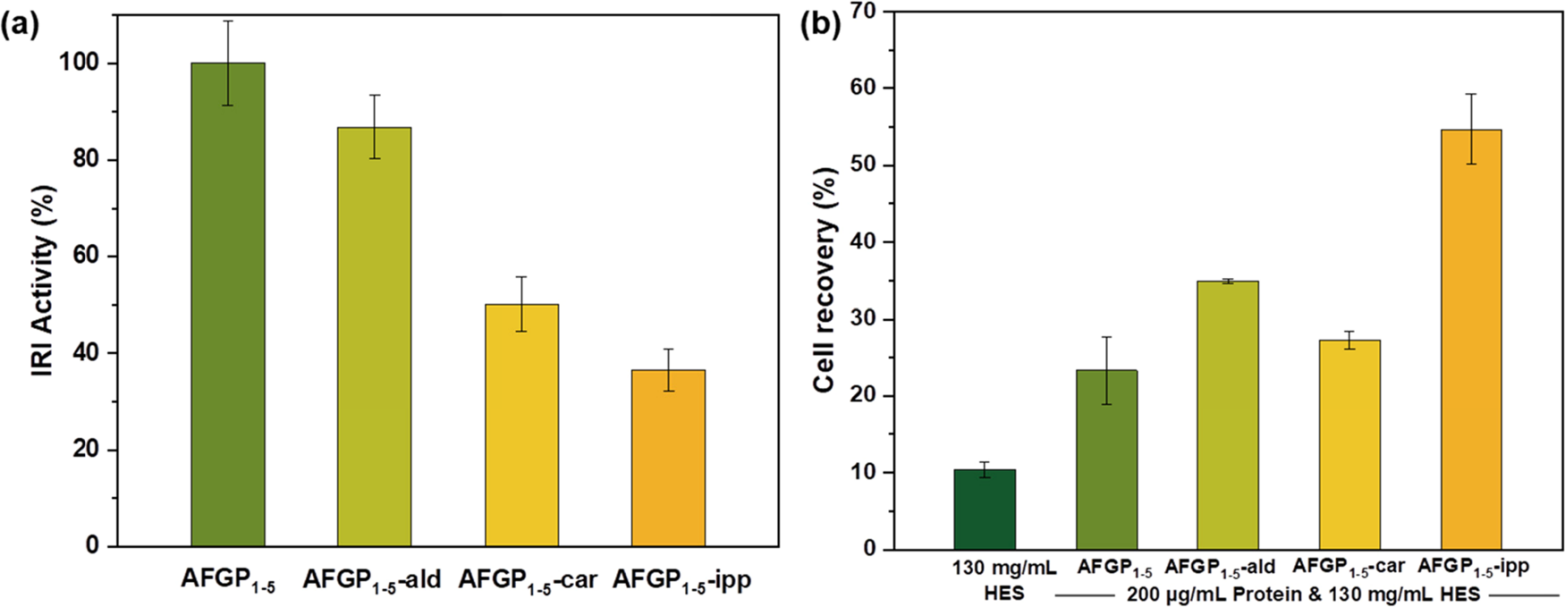
(a) IRI inhibition activity of AFGP_1–5_ and the different variants. The IRI activity of AFGP_1–5_-ald, AFGP_1–5_-car, and AFGP_1–5_-ipp at 2 μg/mL is reduced by ∼13, 50, and 63%, respectively. Reprinted with permission from ref (37). Copyright 2021 ACS Publications.^37^ (b) Effects of AFGP_1–5_ and different variants on the cell recovery of cryopreserved hRBCs in HES solution (130 mg/mL), thawed at 23 °C. Experiments were performed three times, and the error bars represent the standard deviation between the individual measurements.

In order to determine whether there is a correlation between IRI activity and cryopreservation efficacy, three AFGP_1–5_ variants with different IRI and TH activities were investigated. In these variants, the hydroxyl groups of the galactose moieties of the natural AFGP_1–5_ isoforms were modified into AFGP_1–5_-aldehyde (AFGP_1–5_-ald), AFGP_1–5_-carboxyl (AFGP_1–5_-car), and AFGP_1–5_-isopropylidene (AFGP_1–5_-ipp), as described recently.^37,48^ Because of the modifications, the IRI activities of AFGP_1–5_-ald, AFGP_1–5_-car, and AFGP_1–5_-ipp were reduced by 13, 50, and 63%, respectively, relative to native AFGP_1–5_ (Figure 2a).^37^

We investigated the cryopreservation abilities of the variants at 200 μg/mL concentrations in HES (130 mg/mL) because these conditions showed the maximum cryopreservation efficiency for the native AFGP_1–5_. The results are shown in Figure 2b. Despite having lower IRI activity, the hRBC recovery of all three variants was increased. The cell recovery of AFGP_1–5_-ald and AFGP_1–5_-car was increased from 24% for AFGP_1–5_ to 35 and 27%, while AFGP_1–5_-ipp more than doubled cell recovery to 55%. Thus, despite having the lowest IRI activity (37% of native AFGP_1–5_), the AFGP_1–5_-ipp variant enabled the highest hRBC recovery, more than double that of native AFGP_1–5_.

**Figure 3:**
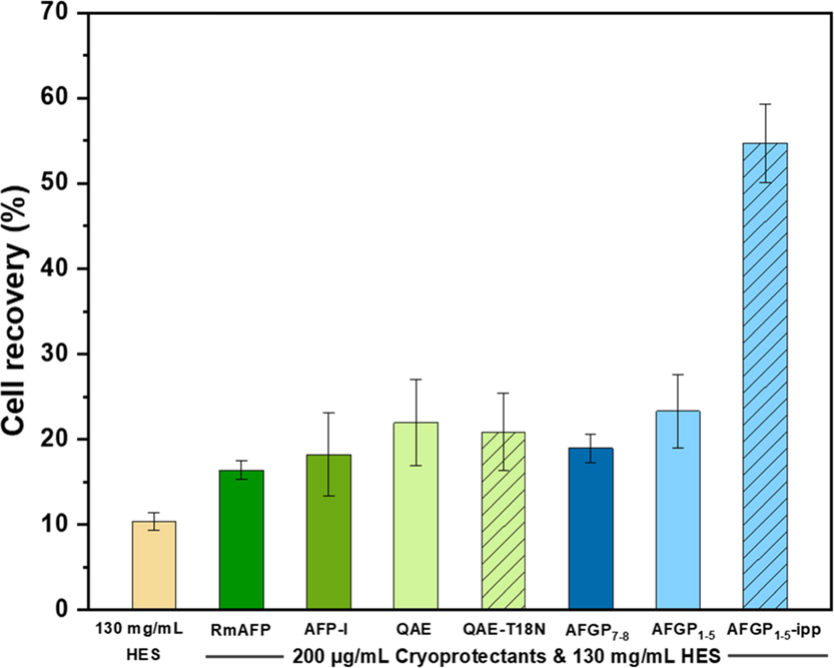
Effect of different AF(*G*)Ps and variants on the cell recovery of cryopreserved hRBC in HES solution (130 mg/mL), thawed at 23 °C. Experiments were performed three times, and the error bars represent the standard deviation between the individual measurements.

Next, we determined the effects of five additional AF(*G*)Ps with varying IRI activities^18^ and compared their ability to improve the survival of hRBCs that were cryopreserved in 130 mg/mL HES solutions (Figure 3). AFGP_7–8_ is an isoform and low-molecular-weight variant of the AFGPs and possesses only ∼60% of the antifreeze activity of the larger AFGP_1–5._^43,49^*Rm*AFP is derived from the beetle *Rhagium mordax* and is considered to be a hyperactive AFP.^14^ QAE is a version of AFP type III with QAE-sephadex-binding,^43,49^ and AFP type I is a moderate AFP derived from winter flounder *Pseudopleuronectes americanus*. For the QAE mutant (QAE-T18N), the threonine 18 residue in the center of the ice-binding site was replaced by asparagine, causing a complete loss of TH activity^50^ and no effect on IRI activity.^51^ We find that the different AF(*G*)Ps show remarkably similar cell recoveries (∼20%) of hRBC despite displaying very different IRI activities (Figure S2). Interestingly, QAE-T18N has the same cell recovery as QAE, revealing that the T18N mutation and the associated loss of TH activity have little effect on the cryopreservation ability of hRBC. Of all investigated AF(*G*)Ps, the AFGP_1–5_-ipp variant shows the highest cell recovery of cryopreserved hRBC. Apparently, the factors influencing the cryopreservation ability of an AF(*G*)P are more complex and not solely dependent on IRI activity.

**Figure 4:**
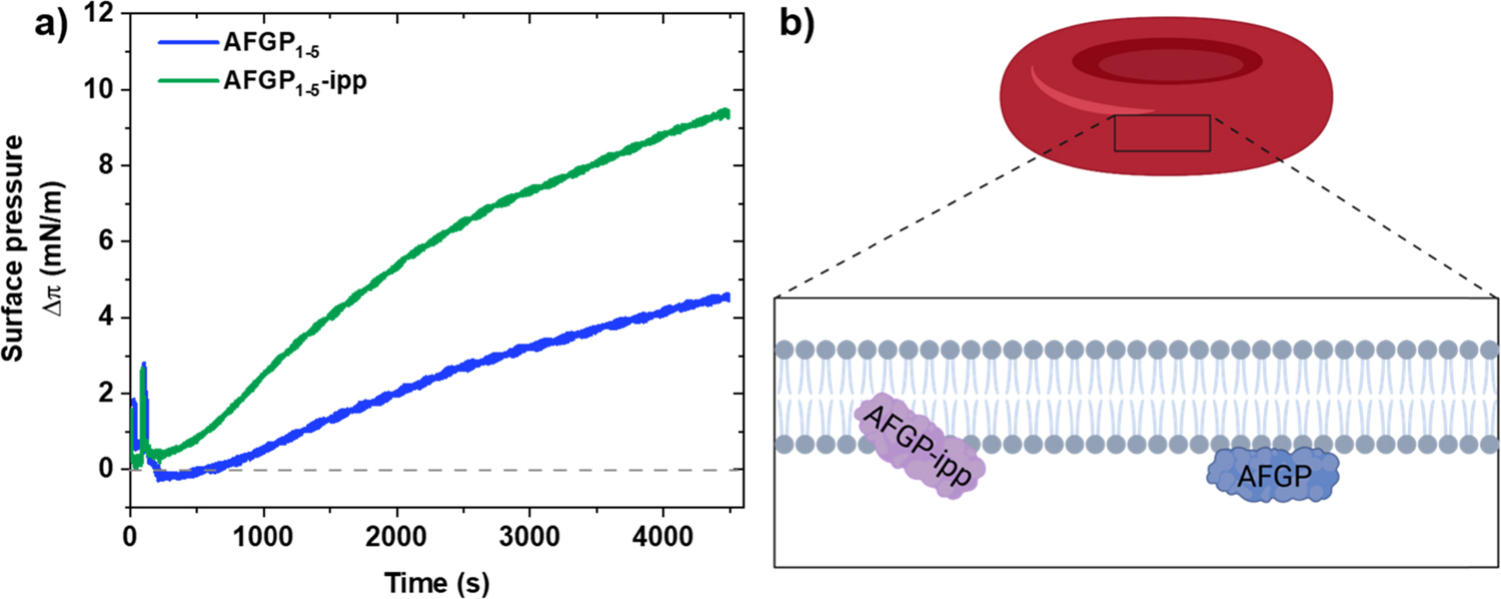
Interactions of AFGP_1–5_ with erythrocyte model membranes. (a) Difference in surface pressure Δπ versus time after the addition of AFGP_1–5_-ipp and AFGP_1–5_ into the PBS subphase of the DOPC/SM/DOPE monolayer. Δπ represents the change in the surface pressure after the addition of AFGP_1–5_ (5 mg/mL) and of PBS buffer. (b) Schematic representation of AFGP_1–5_-ipp and AFGP_1–5_ proteins interacting with an RBC model membrane.

We performed surface pressure experiments of erythrocyte model membranes to further investigate the reason for AFGP_1–5_-ipp’s superior cryoprotective properties. Figure 4 shows the effect of AFGP_1–5_ and AFGP_1–5_-ipp on a model membrane mimicking the erythrocyte outer membrane, consisting of the phospholipid mixture DOPC, SM, and DOPE (45:45:10 (mol %)).^38^ The surface pressure of the model membrane in PBS buffer was set to ∼24 mN/m, typical for a well-ordered, liquid-condensed model membrane.^52^ When proteins were injected under the monolayer, they are allowed to interact with the monolayer. If part of the protein inserts itself in between the lipid molecules or in any way disturbs the lipid packing, the surface pressure will increase. The increase hence suggests that the protein can interact with the monolayer.^41^ We find that the surface pressure increases substantially upon the addition of AFGP_1–5_ and AFGP_1–5_-ipp into the PBS subphase of the DOPC/SM/DOPE monolayer, as shown in Figure 4. This indicates that AFGP_1–5_ and AFGP_1–5_-ipp interact with the monolayer. Δπ represents the difference in the surface pressure after the addition of AFGP_1–5_/AFGP_1–5_-ipp and of the PBS buffer. The increase in surface pressure upon AFGP_1–5_/AFGP_1–5_-ipp injections suggests protein-membrane interactions and possible stabilization of the model membrane. We find the interaction of AFGP_1–5_-ipp with the membrane to be stronger than that of AFGP_1–5_, as indicated by the Δπ of AFGP_1–5_-ipp, which is much higher than that for AFGP_1–5_ (Figure 4a).

## Discussion

The cryopreservation of cells causes cold stress that affects the cell physiology, metabolic activity, and regulation of ion equilibration across membranes. The crystallization of water into ice is further detrimental to tissues, and the process of IRI upon thawing is the major contributor to cell death. We find that the addition of AFGP_1–5_ to a HES cryostorage medium has a statistically significant positive effect on the survival of hRBCs, which is consistent with previous studies on nonglycosylated AFPs.^11,36^ Different AFP classes with varying IRI activities showed very similar cell recovery of hRBC (∼20%), suggesting that IRI activity alone is not sufficient to predict cell recovery after cryopreservation. Interestingly, the chemically modified AFGP_1–5_-ipp variant with additional hydrophobic groups on the galactose moieties promoted cell recovery of hRBC to 55% compared to the 24% of AFGP, while having decreased IRI and TH activity. Apparently, high IRI activity alone is insufficient to explain the beneficial effects of AF(*G*)Ps on hRBC survival after cryopreservation. Alternative mechanisms that explain the positive effect of AF(*G*)Ps on the cold survival of cells involve the protection of cell membranes as they pass through their phase transition temperatures as well as the blockage or alteration of the flow of ions into cells. Tomczak et al. proposed that AF(*G*)Ps insert into membranes through hydrophobic interactions, thereby altering the molecular packing of the acyl chains, resulting in reduced membrane permeability and improved cryopreservation efficiency.^53^ We find that increasing AFGP hydrophobicity by adding an isopropylidene group leads to improved hRBC survival and stronger interaction with an erythrocyte model membrane. We hypothesize that the stabilization of the RBC membranes through AFGP and via hydrophobic interactions is enhanced, owing to the modification. This modification substantially increases hRBC recovery. We suggest that apart from IRI activity, the capabilities to stabilize cell membranes and the cellular location of the cryoprotectants are additional key components for the cell recovery of hRBC after cryopreservation. However, membrane stabilization is an important but not the sole determining factor for cell survival after cryostorage. For example, bovine serum albumin is a membrane stabilizing agent,^54^ but it is usually used as a negative control for cryopreservation study.^55^ Apart from proteins, the lipids, sugars, and amino acids also seem to exert their cryoprotective effect at least partly by stabilizing the plasma membrane.^54,56^ Therefore, the combination of high IRI activity and capabilities to stabilize cell membranes is likely essential for optimizing cell survival after cryostorage.

## Conclusions

Herein, we found a variety of antifreeze proteins that show similar cell recoveries of hRBCs despite having very different IRI activities. In addition, a chemically modified AFGP_1–5_-ipp variant with additional hydrophobic groups on the galactose moiety has decreased IRI activity but showed significantly increased cell recovery compared to AFGP_1–5_. Surface pressure experiments with the AFGP_1–5_-ipp variant showed enhanced interactions with RBC model membranes, indicating that membrane stabilization is another key factor in the survival of cells after cryostorage. We conclude that high IRI activity alone is insufficient to explain the beneficial effects of AF(*G*)Ps hRBC survival. The ability to stabilize cell membranes and the cellular location of the cryoprotectants are also likely crucial for cryopreservation and should be considered for the design of novel synthetic cryoprotectants.

## Supporting Information Available

Influence of different AF(*G*)Ps on the cryopreservation of hRBC; IRI efficacy of various AF(*G*)Ps and their variants (PDF)

## Abbreviations

AFPs: antifreeze proteins
AFGPs: antifreeze glycoproteins
AF(*G*)Ps: antifreeze proteins and glycoproteins
hRBC: human red blood cell
TH: thermal hysteresis
IRI: ice recrystallization inhibition
AFGP_1–5_-ipp: AFGP_1–5_-isopropylidene
AFGP_1–5_-ald: AFGP_1–5_-aldehyde
AFGP_1–5_-car: AFGP_1–5_-carboxyl
PBS: phosphate-buffered saline buffer
HES: hydroxyethyl starch
DOPE: 1,2-dioleoyl-sn-glycero-3-phosphoethanolamine
DOPC: 1,2-dioleoyl-sn-glycero-3-phosphocholine
SM: N-palmitoyl-d-erythrosphingosylphosphorylcho-line
