# Supplementary material for: Ice Recrystallization Inhibition Is Insufficient to Explain Cryopreservation Abilities of Antifreeze Proteins
Source: Biomacromolecules. 2022 Jan 26;23(3):1214–20. doi: 10.1021/acs.biomac.1c01477 (PMC8924859; doi:10.1021/acs.biomac.1c01477)
Supplement: Supplementary file 1 — bm1c01477_si_001.pdf [file bm1c01477_si_001.pdf]

Supporting Information for

Ice Recrystallization Inhibition is Insufficient to Explain Cryopreservation Abilities of Antifreeze Proteins

Yuling Sun[1,2], Daria Maltseva[1], Jie Liu[2], Theordore Hooker II[3], Volker Mailänder[1,4], Hans Ramløv[5], Arthur L. DeVries[6], Mischa Bonn[1], Konrad Meister [1,3]*

[1]Max Planck Institute for Polymer Research, 55128 Mainz, Germany

[2]Institute of Chemistry, Chinese Academy of Sciences, Beijing 100190, P. R. China

[3]University of Alaska Southeast, Juneau, AK, 99801, USA

[4]Dermatology Department, University Medical Center of the Johannes Gutenberg-University, 55131 Mainz, Germany

[5]Roskilde University, 4000 Roskilde, Denmark

[6]University of Illinois at Urbana–Champaign, Urbana, IL, 61801, USA

*Konrad Meister

Email: meisterk@mpip-mainz.mpg.de

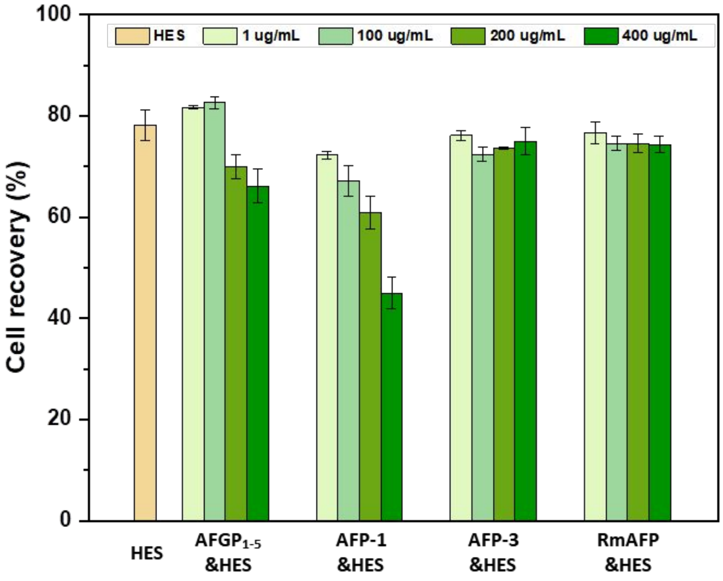

Figure S1. Influence of different AF(G)Ps on the cell recovery of hRBCs cryopreserved in 215 mg/mL HES; thawed at room temperature. The AF(G)Ps were prepared with different concentrations.

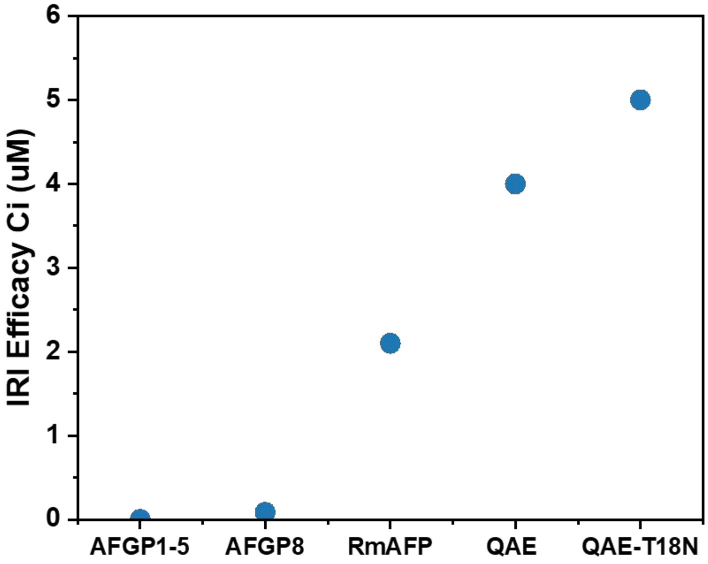

Figure S2. IRI Efficacy (c_i) of various AF(G)Ps and their variants. The c_i of RmAFP is estimated based on the hyperactive insect DAFP with a similar structure and properties [1].

IRI Efficacy (c_i) is defined as the concentration at which the ice recrystallization rate is dominated by the IRI compound [1-3]. AF(G)Ps can show very different IRI activities because of their different IRI efficacies (Figure S2).

Reference

1. Olijve, L. L. C.; Meister, K.; Devries, A. L.; Duman, J. G.; Guo, S.; Bakker, H. J.; Voets, I. K., Blocking rapid ice crystal growth through nonbasal plane adsorption of antifreeze proteins. *Proc. Natl. Acad. Sci. U.S.A.* **2016,** *113*, 3740-3745.
2. Budke, C.; Dreyer, A.; Jaeger, J.; Gimpel, K.; Berkemeier, T.; Bonin, A. S.; Nagel, L.; Plattner, C.; DeVries, A. L.; Sewald, N.; Koop, T., Quantitative Efficacy Classification of Ice Recrystallization Inhibition Agents. *Cryst. Growth Des.* **2014,** *14*, 4285-4294.
3. Olijve, L. L. C.; Oude Vrielink, A. S.; Voets, I. K., A Simple and Quantitative Method to Evaluate Ice Recrystallization Kinetics Using the Circle Hough Transform Algorithm. *Cryst. Growth Des.* **2016,** *16*, 4190-4195.
